# The future of islet transplantation beyond the BLA approval: challenges and opportunities

**DOI:** 10.3389/frtra.2025.1522409

**Published:** 2025-03-07

**Authors:** Yong Wang, James McGarrigle, Jenny Cook, Peter Rios, Giovanna La Monica, Yingying Chen, Wei Wei, Jose Oberholzer

**Affiliations:** ^1^ Clinic of Visceral and Transplant Surgery, University Hospital Zurich, Zurich, Switzerland; ^2^ Faculty of Medicine, University of Zurich, Zürich, Switzerland; ^3^ CellTrans, Inc., Chicago, IL, United States

**Keywords:** islet transplant, type I diabetes, federal food and drug administration, biologics license application (BLA), LANTIDRA

## Abstract

This opinion paper explores the path forward for islet transplantation as a cell therapy for type 1 diabetes, following the Biologics License Application (BLA) approval. The authors review key challenges and opportunities that lie ahead. After a brief overview of the history of human islet transplantation, the paper examines the FDA's regulatory stance on isolated islet cells and the requirements for obtaining a BLA. The authors discuss the significance of this approval and the critical steps necessary to broaden patient access, such as scaling up production, clinical integration, reimbursement frameworks, post-marketing surveillance, and patient education initiatives. The paper highlights that the approval of LANTIDRA as an allogeneic cell transplant for uncontrolled type 1 diabetes marks the beginning of new chapters in improving islet transplantation. The authors emphasize essential areas for development, including advancements in islet manufacturing, optimization of transplant sites, islet encapsulation, exploration of unlimited cell sources, and gene editing technologies. In conclusion, the future of islet transplantation beyond the BLA approval presents challenges and opportunities. While significant regulatory milestones have been reached, hurdles remain. Innovations in stem cell-derived islets, cell encapsulation, and gene editing show promise in enhancing graft survival, expanding the availability of transplantable cells, and reducing the reliance on immunosuppressive drugs. These advancements could pave the way for more accessible, durable, and personalized diabetes treatments.

## Brief history of human islet transplant

**Figure 1:**
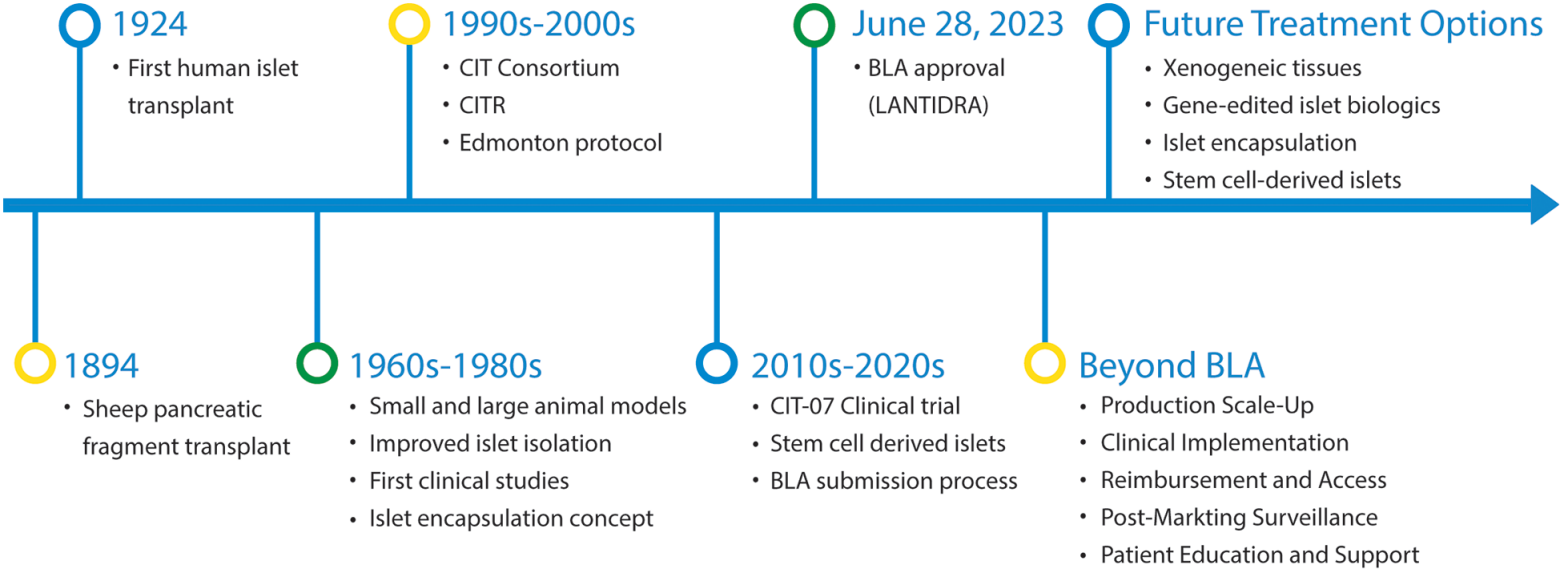
Historical timeline of significant events in the process of human islet transplant and beyond BLA approval.

The potential of islet transplantation as a treatment for Type 1 diabetes (T1D) was first proposed over a century ago (Figure 1). Pioneers like Dr. Watson-Williams at the Bristol Infirmary in England in 1894 and English surgeon Dr. Charles Pybus in 1924 explored this concept in clinical practice. The initial attempts at islet transplantation were mainly experimental, with minimal to no success.

The modern era of islet transplantation is largely credited to Dr. Paul Lacy, who introduced a collagenase-based method for isolating rat islets and developed animal transplant models in the 1960s (1, 2). Since then and up to the 1980s, further improvements in human islet isolation were achieved, but translating these to clinical practice remained challenging (3–6).

A breakthrough occurred in the late 1990s with the development of the Edmonton Protocol (7), which combined improved islet isolation methods, advanced immunosuppressive therapies, and the use of the portal vein as the specific transplant site. This protocol significantly enhanced transplant outcomes and led to successful cases of insulin independence in brittle T1D patients, characterized by severe hypoglycemia and glycemic instability despite insulin therapy (7, 8). Since then, human islet transplantation has continued to advance with better techniques for the isolation of islets, novel immunosuppressive drugs, and anti-inflammation approaches to assist in the protection of transplanted islet grafts. Worldwide clinical studies have demonstrated that islet transplantation can significantly improve glycemic control and achieve insulin independence for patients presenting with brittle T1D (9–15). However, despite these clinical successes, islet transplantation has remained a niche therapy due to a variety of challenges, including limited donor supply, immune rejection, and need for lifelong immunosuppressive therapy.

## The biologics license application (BLA)

Unlike the European Union, Canada, Australia, Japan, and some other countries, a significant regulatory challenge exists in the United States (US) to expand islet transplantation as a standard therapy for T1D patients. Given the significant manipulations needed to safely and reproducibly isolate islets from a donor pancreas, allogeneic islets are considered a biological drug in the US. Consequently, a Biologics License from the Food and Drug Administration (FDA) is required for its application outside clinical trials. The FDA's “Considerations for Allogeneic Pancreatic Islet Cell Products” in 2008 (Docket Number: FDA-2008-D-0293) and “Guidance for Human Somatic Cell Therapy and Gene Therapy” in 2009 (Docket Number: FDA-2009-D-0132-0016) are two important documents that guide allogeneic pancreatic islet cell products for the treatment of patients with T1D. The guidance covers several key areas (Table 1):

**Table 1:** The guidance of the biologics license application (BLA).

Key regulatory aspects	Guidance and considerations
Product characterization	Source: islet cells from screened deceased donors Isolation: validated, standardized methods and procedures Quality Control: identity, purity, potency, safety, viability, functionality.
Manufacturing controls	Consistency: reproducible product quality Islet Storage Conditions: culture and transport Contamination Prevention: avoid microbial contamination.
Preclinical studies	Animal Models: utilized to test safety and efficacy.
Immunogenicity	Assess immune response and risk of rejection.
Clinical studies	Design: evaluate safety and efficacy while controlling key variables Endpoints: insulin independence and glycemic control Monitoring: adverse events, complications, and rejection.
Post-approval	Post-market Safety Reporting: adverse experience and lot distribution reports.
Regulatory pathways	Submission of detailed trial data and information regarding manufacturing and product characterization.

Hereafter, several publications on the FDA regulatory policies have indicated that although significant progress has been made in standardizing donor organ acceptance, process controls, and product release criteria, key issues of the safety, purity, efficacy, and potency of islet products must be addressed before a BLA can be submitted (16–19).

The BLA process ensures the following (Table 2):

**Table 2:** The process of the biologics license application (BLA).

Evaluation domains	Evaluation criteria and considerations
Safety and efficacy	Verification of the safety and effectiveness of islet transplants.
Manufacturing quality	Assurance of consistent quality and purity of islet cells.
Immune response	Assessment of potential immune reactions induced by islet cells and necessary interventions.
Long-term benefits and risks	Evaluation of long-term outcomes of islet transplants.
Scientific rigor	Comprehensive clinical trials and strong evidence supporting the procedure.

To summarize, Biologics License approval, consistent with other biological therapies, ensures that islet transplants are safe, effective, and adhere to rigorous regulatory standards.

On June 28, 2023, a significant milestone was achieved when CellTrans Inc. received a biological license (Health and Human Services License No. 2213) from the FDA for the manufacture of LANTIDRA (donislecel-jujn) in the US (https://www.fda.gov/news-events/press-announcements/fda-approves-first-cellular therapy-treat-patients-type-1-diabetes). LANTIDRA is an allogeneic pancreatic islet cellular therapy indicated for the treatment of adults with T1D who are unable to approach target HbA1c because of current repeated episodes of severe hypoglycemia despite intensive diabetes management and education. LANTIDRA is used in conjunction with concomitant immunosuppression.

In the FDA press release published on the day LANTIDRA was approved, Peter Marks, M.D., Ph.D., Director of the FDA's Center for Biologics Evaluation and Research, stated: “Severe hypoglycemia is a dangerous condition that can lead to injuries resulting from loss of consciousness or seizures. Today's approval, the first-ever cell therapy to treat patients with Type 1 diabetes, provides individuals living with Type 1 diabetes and recurrent severe hypoglycemia an additional treatment option to help achieve target blood glucose levels”.

FDA approval marked a turning point for islet transplantation, setting the stage for increased access to the therapy in the US. It also introduced a standardized regulatory framework, to ensure that future advancements in islet transplantation follow established safety and quality guidelines.

The FDA's decision to regulate islet transplants as a biologic drug, requiring a Biological License, has sparked debate within the medical and research communities. Points of contention include the regulatory burden, patient access, the classification of islets, and the impact on research and innovation (20). Despite these concerns, the FDA's classification of islets as a biologic drug reflects its commitment to patient safety through rigorous oversight. The agency remains firm that these measures are necessary to ensure high standards of safety and efficacy, while continuing to work with stakeholders to streamline the process.

## Next steps for islet transplantation post biologics license approval

To ensure islet transplantation can be a viable treatment option for more T1D patients following FDA approval, challenges related to islet supply, long-term efficacy, and cost must be addressed.

### Production scale-up

Scaling up production is critical to ensure a reliable supply of human islets. This involves the establishment of cGMP (current Good Manufacturing Practice) facilities for islet manufacture, while strictly adhering to regulatory and safety standards. This ensures that the biological product is consistently produced with the same quality, potency, and purity, reducing the risk of contamination and minimizing variability between batches. The implementation of automation and efficiency improvements in the islet manufacture process could help reduce production costs while maintaining high product quality.

### Clinical implementation

It is important to establish qualified transplant centers where the procedures can be performed, including training transplant surgeons and medical staff on defined human islet transplant protocols and procedures. In the participating transplant centers, screening procedures and defined selection criteria have to be implemented to identify patients who are the most suitable candidates, focusing on individuals who present with brittle T1D that have been well-established in the field of islet transplant (13, 21). To qualify, patients must be 18 or older, have had T1D for at least five years, and have a BMI below 27. They should also have experienced at least one severe hypoglycemic episode in the past year and suffer from impaired awareness of hypoglycemia despite ongoing insulin therapy, intensive diabetes management, and education. Furthermore, patients must be free from major cardiovascular, respiratory, liver, or brain conditions and should not have any active infections.

### Reimbursement and access

Negotiating reimbursement with health insurers is critical to increase patient access. Given the high costs associated with islet transplantation and post-transplant care (e.g., immunosuppressive therapy), working with payors to secure coverage is essential for the widespread adoption of islet transplantation. Coverage by both government payors (e.g., Center for Medicare and Medicaid Services) and private payors have to be secure to enable unrestricted access to allogeneic islet cell therapy for the small population of brittle T1D. At the time of writing, most private payors in the US have included Lantidra under their covered benefits for brittle T1D (https://www.uhcprovider.com/content/dam/provider/docs/public/policies/clinical-guidelines/transplant-review-guidelines-solid-organ-transplantation.pdf).

### Post-Marketing surveillance

The FDA requires safety monitoring of LANTIDRA (i.e., pharmacovigilance) per 21 CFR 600.80 and the submission of lot distribution reports per 21 CFR 600.81. Ongoing monitoring and reporting of adverse events, complications related to the islet transplant and the immunosuppressive therapies used to prevent rejection is essential for patient safety.

### Patient education and support

Comprehensive patient education programs should be developed to inform potential recipients about the risks and benefits of the procedure, as well as the need for lifelong follow-up and medication adherence. Support networks and programs can help transplant recipients manage their health, including guidance on managing immunosuppression and monitoring for signs of rejection or complications.

The success of human islet transplantation will depend on overcoming challenges related to supply, cost, long-term efficacy, and patient access. These steps are part of a broader effort to make islet transplantation more widely accepted and available as a treatment option for T1D patients.

### Challenges and opportunities beyond the BLA

**Figure 2:**
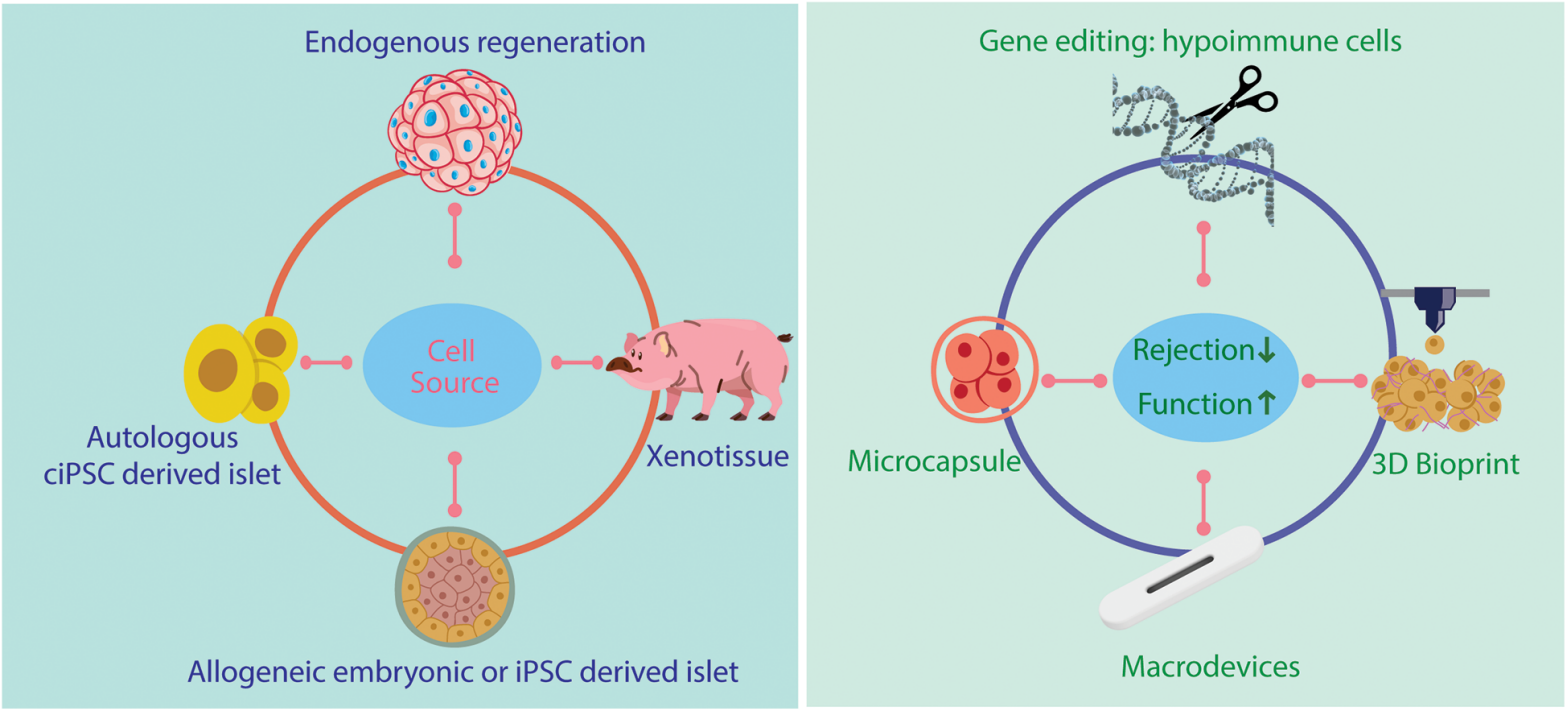
Future therapy of human islet transplant.

While the FDA approval represents a major regulatory milestone, it does not address several critical challenges that impede the widespread adoption, especially on how to avoid immunosuppressant and solve limited islet sources (Figure 2).

## Islet manufacturing

After the breakthrough of the Edmonton Protocol, significant progress has been made in islet isolation techniques (22–26). The advances have been largely driven by the need for more consistent and higher-quality islet yields, improved enzyme formulations, and enhanced organ preservation methods. However, not every donated pancreas delivers sufficient amounts of islets, defined by the minimal post-purification islet mass needed for an islet transplant to achieve clinically relevant benefits (in general a minimum of 5,000 IEQ/Kg patient body weight is needed to confer improved metabolic control).

Enzyme blends for pancreas tissue digestion during manufacturing can significantly affect islet yield and quality (27, 28). While great progress has been achieved in the manufacturing of those enzyme blends, batch to batch variabilities are still a challenge in achieving product consistency in islet manufacturing.

Product characterization has been another area of contention in the field. Product characterization aims to ensure proper release criteria to reduce the risk of poor-functioning islet grafts. New developments in digital image analysis and microfluidic Islet-On-Chip technology may better assist researchers in developing real-time assessment methods for islet health and functionality, ensuring only the most viable and functional islet preparations are selected for transplantation to further improve the chances of long-term success (29–35).

In our own experience, applying stringent cGMP principles with standardization of the manufacturing process has led to greater consistency in islet yield and quality. This should lead to broader clinical application, though there is still room for further improvements. Future work may focus on the automation of the actual islet isolation process with better control over variables such as enzyme temperature and digestion time.

Recent advances in organ perfusion techniques for islet transplantation, including hypothermic and normothermic machine perfusion, have shown promising results (36–38). These methods are designed to improve organ preservation prior to its use in islet manufacture, as well as increase islet viability for transplantation. Hypothermic machine perfusion has demonstrated benefits in preclinical studies, particularly in extending preservation times, which is especially valuable when using organs from donors with circulatory death (DCD). Normothermic machine perfusion is gaining momentum as a method to assess and enhance graft viability before transplantation, allowing real-time evaluation of the pancreas and reducing tissue injury during the islet isolation process. These organ perfusion techniques have great potential to improve islet transplant outcomes by enhancing donor organ quality and expanding the donor pool. Future studies are necessary to validate these promising preclinical findings.

At present, transplants are only performed with freshly isolated islets. The logistics involved in handling of fresh tissue with a limited shelf life present a significant challenge for wider geographical access to islet transplantation. Historically, the recovery yield of cryopreserved islets had been frustrating low, despite of decade of intense research. With recent progress in islet cryopreservation this field may need to be revisited and tested in the clinical context (39, 40). This would enable more flexible use of available donor organs and better matching of islet quality to recipient needs, potentially reducing waste and improving transplant success rates.

Last, but not least, the main limiting factor in the manufacture of islets is the availability of human donor pancreases. Increasing awareness regarding the importance of organ donation through public health campaigns have led in recent years to an increase in organ donations. Continued public health efforts to encourage individuals to become registered donors can help alleviate the shortage of available organs for islet isolation.

Each of these strategies holds promise, and collectively, could help mitigate the donor supply shortage that will become more acutely once islet transplantation becomes more accessible post-BLA approval of LANTIDRA.

### Transplant site

At present islet grafts are delivered intrahepatically via portal vein access. Intraportal islet transplantation is an established technique that provides immediate blood supply and has shown to be reasonably safe and with good clinical success in reversing severe hypoglycemia and achieving insulin independence in the majority of recipients. However, this site is also associated with drawbacks, including significant, immediate islet loss via an instant blood-mediated inflammatory reaction (IBMIR) (41, 42). Other transplant sites have been explored such as the omentum, gastric submucosa, and muscle tissues (43–48), though, they have not yet shown superiority to the portal route. Despite promising case series, further larger-scale clinical studies are needed to validate new transplant sites and determine their efficacy in improving transplant outcomes.

### Islet encapsulation

Even with the use of immunosuppressive regimens, immune rejection of transplanted islets remains a concern. The need for lifelong immunosuppression to prevent rejection exposes patients to significant side effects, including an increased risk of infection and cancer. In the last four decades, enormous efforts have been underway to protect isolated islets from immune responses through islet encapsulation.

The concept of islet microencapsulation originated in the 1970s, with early research focusing on alginate, a biocompatible polymer, to form semi-permeable membrane that allows insulin and nutrients to pass through while blocking immune cells and antibodies (49). In the 1980s and 1990s, advancements in alginate-based encapsulation techniques, such as the microencapsulation of islets in alginate-poly-L-lysine shells, demonstrated promise (50–52). However, fibrosis (formation of scar tissue around the capsules) remained a significant challenge, limiting long-term islet function.

By the 2000s, clinical trials using encapsulated islets began, demonstrating short-term protection but often failing due to capsule surface fibrotic overgrowth, which compromised the islet's oxygen and nutrient supply (53, 54). Despite challenges, these early clinical efforts provided valuable insights that continue to guide modern research.

From the 2010s onward, new encapsulation strategies have emerged, focusing on reducing fibrosis and improving islet survival. These include advanced biocompatible materials (55–58), nanotechnologies such as ultra-thin coatings designed to be more biocompatible/durable (59, 60), and new transplant sites (61, 62).

Macrodevices are implantable devices that house clusters of islets. One notable example is the TheraCyte device, which uses a semi-permeable membrane to encase the islets. It has shown some success in both preclinical and clinical trials, particularly in improving long-term islet survival and function (63–66). Another promising macrodevice is the Beta-O2 device, which features an oxygen reservoir that sustains islet function by providing a steady oxygen supply. This method has demonstrated superior outcomes in maintaining islet viability and function over longer periods compared to standard encapsulation methods (67–69). Implantable scaffolds, made from biocompatible materials, encourage vascularization around the islets, enhancing their survival and insulin production. Alginate fiber for islet encapsulation is an innovative approach that utilizes modified alginate formulations and combination materials to improve biocompatibility and reduce immune responses (70). These advancements have shown promising preclinical results, indicating potential benefits for islet transplant outcomes.

After the FDA approval of LANTIDRA, interest in islet encapsulation technology has intensified as a complementary solution to eliminate the need for immunosuppression, particularly with the development of more biocompatible and durable polymers like modified alginates and advanced hydrogels, focusing on optimized capsule size, shape, and mechanical properties, as well as addressing issues like fibrotic overgrowth. One novel approach is nanotechnology, where ultra-thin coatings provide more precise control over permeability and help evade immune detection.

For macrodevices, integrating oxygen-releasing agents and nanostructured membranes into encapsulation materials enhances oxygen delivery, which is crucial for long-term islet function (67, 71). Newer methods also co-encapsulate islets with anti-inflammatory or pro-regenerative factors, creating a localized immunomodulatory environment that protects against immune attack and promotes better tissue integration (72). More recently, 3D printing technology has allowed for precise control over scaffold architecture, including features such as pore size and connectivity, which are critical for nutrient and oxygen diffusion, as well as device strength (73–75).

Ongoing clinical trials are exploring advanced polymers, oxygenating agents, bioengineered scaffolds, and xenogeneic tissues, which could significantly improve the availability and effectiveness of islet transplantation without the need for lifelong immunosuppression.

### Limited islet cell sources

Islet transplantation remains dependent on a limited supply of deceased donor organs, making it inaccessible for many patients. The shortage of viable donor pancreas has led to a significant gap between the number of patients that can benefit from the therapy and the availability of islets. The shortage of suitable pancreas donors remains as a significant obstacle to islet transplantation.

Use of Extended Criteria Donors (ECDs) and Donors after Cardiac Death (DCD) who do not meet standard criteria due to age, BMI, or medical history are now being considered. Advances in enzyme digestion protocols and preservation techniques, such as organ perfusion, have made it possible to recover viable islets from previously unsuitable organs.

Pig islets can be used for human transplantation due to their physiological similarity with human islets. Using them addresses the shortage of human donor organs by providing a more abundant and scalable source, offering potentially shorter wait times for administration through consistent availability.

Genetically modified pigs are being developed to reduce immune rejection of islet transplants (76–79), including expressing human HLA molecules to lower immune recognition, deleting the alpha-galactosidase enzyme to avoid immune responses triggered by alpha-gal epitopes, and inserting genes that produce immunomodulatory proteins like CTLA4-Ig or PD-L1. Additionally, pigs can be engineered to express human complement regulators, such as CD46 or CD55, to protect islet cells from complement-mediated damage (80). Gene editing techniques like CRISPR/Cas9 are also used to knock out inflammatory genes or introduce genes that promote immune tolerance (81). Initial preclinical trials using naïve pig islets and genetically modified pig islets have shown encouraging results, particularly in non-human primates. These studies have demonstrated that pig islets can survive and function in primates for extended periods, especially when combined with immunosuppressive therapy or encapsulation (82–84).

Clinical trials testing pig islets in humans began as early as 2009 in New Zealand by Living Cell Technology. The results demonstrated some positive outcomes, including improved blood sugar control and reduced insulin requirements. However, these trials did not achieve long-term islet graft function or complete insulin independence (85, 86). Several other studies have similar clinical outcomes (87, 88).

In the US, an Investigational New Drug (IND) must be filed with the FDA, including detailed information about the source of the animal tissue, the genetic modifications made to reduce immune rejection, preclinical safety and efficacy data, and the design of the proposed clinical trial. Recently, an encapsulated pig islet IND has been filed and approved for islet transplantation (ClinicalTrials.gov NCT06575426). Clinical results are expected to be released in the course of 2025.

Xenotransplantation faces significant regulatory challenges, particularly related to biosafety concerns. One major issue is the risk of transmitting porcine endogenous retroviruses (PERVs) from pigs to humans, although studies suggest minimal risk with genetically modified pigs. Regulatory agencies, such as the FDA, mandate rigorous safety protocols and continuous monitoring for xenotransplantation trials. The future of xenotransplantation will depend on advancements in genetic modifications, improved immune protection strategies, and addressing ethical and safety concerns. If these challenges are met, xenotransplantation may provide a scalable and reliable source of islets for treating patients with T1D.

Since the 1970s, researchers have been investigating regenerative approaches by differentiating human pluripotent stem cells (hPSCs) and induced pluripotent stem cells (iPSCs) into functional insulin-producing beta cells by mimicking developmental cues from embryogenesis.

In the 1990s, early research focused on differentiating embryonic stem cells (ESCs) into insulin-producing beta cells (69, 89). Progress accelerated with the introduction of induced pluripotent stem cells (iPSCs) in 2007 (90), allowing for the generation of beta- cells from adult cells. Throughout the 2010s, advancements in differentiation protocols improved the functionality of these cells, closely mimicking natural insulin-producing beta -cells that could normalize blood sugar levels in diabetic mice (91–95). Future research will focus on enhancing cell survival, glucose responsiveness, and long-term integration, aiming to create a reliable and scalable source of insulin-producing cells for diabetes treatment.

Non-human primates (NHPs) are frequently utilized in preclinical models due to their close physiological resemblance to humans, providing a valuable system for studying diabetes treatments. Successful studies have shown that human pluripotent stem cell (hPSC)-derived islets can enhance glycemic control and alleviate diabetes symptoms in NHPs (62, 96), which bolsters the potential of hPSC-derived islets as a promising therapy for diabetes. Future research will focus on refining transplantation protocols, addressing immunological concerns, and further optimizing these therapies before advancing to human clinical trials, as well as assessing potential teratoma risk.

Regarding clinical studies with stem cell-derived islets, several companies are leading the way in advancing toward clinical trials. ViaCyte, a California biotech firm, is testing PEC-Direct (Pancreatic Endocrine Cells Direct), a therapy that implants stem cell-derived pancreatic progenitors in a semi-permeable device under the skin (ClinicalTrials.gov NCT03163511). These cells mature into insulin-producing beta-cells, potentially reducing or eliminating the need for external insulin (97, 98). In February 2022, ViaCyte and CRISPR Therapeutics announced phase I clinical trials of VCTX210, a hESC-based therapy for T1D without the need for immunosuppression. The CyT49 human embryonic stem cell (hESC) line is genetically engineered to lack the beta-2 microglobulin (B2M) gene, preventing the expression of major histocompatibility complex (MHC) class I molecules, and to express a transgene encoding programmed death ligand 1 (PD-L1) for protection against CD8+ cytotoxic T-cell attack. These modifications enhance immune evasion, making CyT49 a promising candidate for cell-based therapies such as islet transplantation, potentially reducing the need for long-term immunosuppression and improving graft survival (99, 100).

In 2021, Vertex Pharmaceuticals began a clinical trial using VX-880, beta cells differentiated from human pluripotent stem cells., with immunosuppression. These cells are engineered to function similarly to natural beta cells, responding to blood glucose levels by secreting insulin. At the ADA's 84th Scientific Session (2024), Vertex presented that after a single VX-880 infusion, all twelve patients showed islet engraftment and glucose-responsive insulin production by day 90. All had improved glycemic control, reducing or eliminating insulin use. The three patients with over a year of follow-up met the primary endpoint of severe hypoglycemic episode (SHE) elimination with HbA1c<7.0% and the secondary endpoint of insulin independence. VX-880 was well tolerated with mostly mild to moderate adverse events, and no serious events related to the treatment; two deaths occurred but were unrelated to VX-880.

VX-880 represents a significant advancement in diabetes research, with the potential to profoundly affect the management of T1D. Ongoing and future trials will be essential to assess the therapy's viability and its potential for long-term benefits to patients. Currently, Vertex is conducting a Phase 1 clinical trial to evaluate the safety, tolerability, and preliminary efficacy of the encapsulated stem cells in a small group of participants.

A recent study reported 1-year outcomes for a patient with T1D who underwent autologous transplantation of chemically induced pluripotent stem-cell-derived islets (CiPSC islets) beneath the abdominal anterior rectus sheath with standard immunosuppression (101). The patient achieved sustained insulin independence within 75 days. By month 4, the patient's time-in-target glycemic range had increased from a baseline of 43.18%–96.21%, stabilizing at over 98% with an HbA1c of approximately 5%. Two additional patients have also been transplanted, with results expected in 2025. This study marks significant progress toward personalized cell therapy for T1D using CiPSCs.

Additionally, several ongoing national and international clinical trials are listed on ClinicalTrials.gov, although no transplant outcomes have been reported to date.

Some research efforts combine stem cell-derived islet transplantation with novel immunotherapies designed to retrain the immune system to tolerate beta cells, which has been well-reviewed elsewhere (102). This approach could enhance the durability of the grafts while minimizing the need for immunosuppression. Immune modulation strategies aim to create a more favorable environment for the transplanted beta cells, preventing autoimmune destruction.

While stem cell studies have demonstrated significant benefits for glycemic control, several challenges persist, including immune rejection, long-term graft function, *in vivo* cell maturation, efficacy, and safety concerns, and high manufacturing costs. Despite promising results in ongoing research, these hurdles need to be addressed before this approach can become a standard diabetes treatment.

### Gene editing and CRISPR technology

Gene editing tools, such as CRISPR-Cas9 (Clustered regularly interspaced short palindromic repeats and CRISPR-associated protein 9) are being utilized to enhance the safety and efficacy of islet biologics and stem cell-derived islets. CRISPR-Cas9 targets specific DNA sequences, creating double-strand breaks to allow precise genetic modifications. Its precision, efficiency, and versatility have revolutionized genetics, research, and medical practice. The genetic makeup of these cells aims to reduce immune responses (immune evasion), improve their functionality, and increase the efficiency of differentiating them into functional beta cells.

Traditional immune-evasion strategies are designed to improve graft survival and function. These include immunoisolation devices, immunosuppressive drugs, and tolerance induction techniques.

More recently, there has been growing interest in using gene editing for immune evasion, as it offers new potential for improving transplant outcomes. Deleting genes responsible for expressing MHC class I molecules and co-stimulatory signals made islets less recognizable to the immune system. For example, one hPSC study in which the majority of the polymorphic human leukocyte antigens (HLAs), the main drivers of allogeneic rejection, are deleted and tested in *in vivo* humanized mouse models, showing that these gene manipulations significantly reduce NK cell activity and T-cell-mediated alloimmune response against hPSC-derived islet cells (103).

One study engineered hypoimmune (B2M/, CIITA/, CD47+) primary rhesus macaque pseudo-islets and transplanted them into a fully allogeneic, immunocompetent, diabetic cynomolgus monkey without immunosuppression, showing that the islet grafts quickly normalized c-peptide and glucose. The recipient monkey became insulin-independent long-term without showing any side effects (104). Another study from the same group demonstrated that rhesus macaque hypoimmune pluripotent (HIP) stem cells survived for 16 weeks without immunosuppression by depleting HLA class I and II molecules and overexpressing CD47 (B2M^−/−^CIITA^−/−^CD47^+^) in a fully immunocompetent allogeneic rhesus macaques recipient, whereas allogeneic wild-type cells were vigorously rejected (105).

One study found that targeting human leukocyte antigens (HLAs) and PD-L1 alone does not provide adequate protection against xenograft or allograft rejection of SC islets (stem cell-derived islet cells). To enhance protection, the researchers genetically engineered SC-islet cells to secrete IL-10, TGF-β, and a modified form of IL-2, which promote a tolerogenic local microenvironment by recruiting regulatory T cells to the islet grafts. These cytokine-secreting human SC-β cells demonstrated resistance to rejection and successfully reversed diabetes for up to 8 weeks in non-obese diabetic (NOD) mice (106).

In summary, gene editing and immune evasion represent a new horizon for islet transplantation, improving graft acceptance, reducing reliance on immunosuppressive drugs, and addressing donor shortages. This approach marks a significant advance toward personalized and regenerative medicine for diabetes, with the potential to transform future treatment options.

## Regulatory T cells (Tregs) in islet transplant

Administering regulatory T cells (Tregs), capable of suppressing immune responses, or expanding these cells ex-vivo and reintroducing them to the patient, is a promising approach. Current researchers are actively exploring methods to enhance the efficacy and stability of Tregs in managing immune responses, which has been systemically well- reviewed (107).

## Conclusions

The future of islet transplantation beyond the Biologics License presents significant challenges and promising opportunities. The path toward the widespread clinical adoption of LANTIDRA remains complex and lengthy. As of this writing, LANTIDRA has been covered by most private insurers in the U.S. for patients with brittle T1D. Additionally, the FDA has recently approved LANTIDRA's shipping protocols for the shelf life of LANTIDRA up to 48 h, facilitating broader distribution. On November 25, 2024, the University of Illinois Health in Chicago initiated LANTIDRA therapy in partnership with CellTrans. Throughout 2024, CellTrans engaged in extensive discussions with regional and national islet transplant programs, aiming to launch a multicenter implementation by 2025.

Despite these advancements, other key challenges remain, including but not limited to (1) Pancreas Allocation & UNOS Compliance: Ensuring organ availability and adherence to United Network for Organ Sharing (UNOS) guidelines. (2) Islet Isolation Facilities: Expanding the number of qualified centers to meet the growing demand for islet isolation. At this writing, Celltrans only receives pancreas organs from local OPO and allocates for T1D patients at the University of Illinois Health in Chicago. it is an urgent task to establish additional isolation facilities nationwide to prevent potential pancreatic ischemia-reperfusion injuries and efficiently utilize pancreas organs, which is expected between 2025 and 2026. (3) Islet transplant after kidney transplant is a promising therapy, it addresses two critical issues: restoring glycemic control and protecting the transplanted kidney from the damaging effects of uncontrolled diabetes. LANTIDRA, however, is not labeled by the FDA for islet transplant after kidney transplant in the US. Future evidence has to be provided to FDA approval for indication and usage (https://www.fda.gov/media/169920/download). (4) Patient-Specific Needs & Access: Refining eligibility criteria, accommodating individual patient needs, and ensuring equitable access across diverse populations. Addressing these challenges will require years of collaboration among clinicians and healthcare systems to refine protocols and establish the necessary infrastructure. This long and demanding process will be filled with obstacles before LANTIDRA can fully transform clinical practice.

Furthermore, while regulatory milestones have been met, critical issues persist, such as limited donor availability, immune rejection, the need for lifelong immunosuppression, and inconsistent transplant outcomes. However, emerging innovations in stem cell-derived islets, cell encapsulation, and gene editing offer hope for overcoming these barriers. These advancements have the potential to improve graft survival, increase the availability of transplantable cells, and reduce dependence on immunosuppressive therapies, ultimately paving the way for more accessible, durable, and personalized diabetes treatments in the future.
